# The *Bicoid* Class Homeodomain Factors *ceh-36/OTX* and *unc-30/PITX* Cooperate in *C*. *elegans* Embryonic Progenitor Cells to Regulate Robust Development

**DOI:** 10.1371/journal.pgen.1005003

**Published:** 2015-03-04

**Authors:** Travis Walton, Elicia Preston, Gautham Nair, Amanda L. Zacharias, Arjun Raj, John Isaac Murray

**Affiliations:** 1 Department of Genetics, Perelman School of Medicine, University of Pennsylvania, Philadelphia, Pennsylvania, United States of America; 2 Department of Bioengineering, University of Pennsylvania, Philadelphia, Pennsylvania, United States of America; 3 Penn Genome Frontiers Institute, University of Pennsylvania, Philadelphia, Pennsylvania, United States of America; University of Cambridge, UNITED KINGDOM

## Abstract

While many transcriptional regulators of pluripotent and terminally differentiated states have been identified, regulation of intermediate progenitor states is less well understood. Previous high throughput cellular resolution expression studies identified dozens of transcription factors with lineage-specific expression patterns in *C*. *elegans* embryos that could regulate progenitor identity. In this study we identified a broad embryonic role for the *C*. *elegans OTX* transcription factor *ceh-36*, which was previously shown to be required for the terminal specification of four neurons. *ceh-36* is expressed in progenitors of over 30% of embryonic cells, yet is not required for embryonic viability. Quantitative phenotyping by computational analysis of time-lapse movies of *ceh-36* mutant embryos identified cell cycle or cell migration defects in over 100 of these cells, but most defects were low-penetrance, suggesting redundancy. Expression of *ceh-36* partially overlaps with that of the *PITX* transcription factor *unc-30*. *unc-30* single mutants are viable but loss of both *ceh-36* and *unc-30* causes 100% lethality, and double mutants have significantly higher frequencies of cellular developmental defects in the cells where their expression normally overlaps. These factors are also required for robust expression of the downstream developmental regulator *mls-2/HMX*. This work provides the first example of genetic redundancy between the related yet evolutionarily distant *OTX* and *PITX* families of bicoid class homeodomain factors and demonstrates the power of quantitative developmental phenotyping in *C*. *elegans* to identify developmental regulators acting in progenitor cells.

## Author Summary

Animals develop as one initial cell, the fertilized egg, repeatedly divides and its progeny differentiate, ultimately producing diverse cell types. This occurs in large part by the expression of unique combinations of regulatory genes, such as transcription factors, in precursors of each cell type. These early factors are typically reused in precursors of different cell types. The nematode worm *Caenorhabditis elegans* is a powerful system in which to identify developmental regulators because it has a rapid and reproducible development, yet it shares most of its developmental regulators with more complex organisms such as humans. We used state-of-the-art microscopy and computer-aided cell tracking methods to identify the developmental role of worm homologs of the *OTX* and *PITX* genes, whose human homologs play a role in the development of the brain, eye, and pituitary among other tissues. We identified broad roles for *OTX* in regulating development for many distinct cell types including muscles, neurons and skin, and found a redundant role for both *OTX* and *PITX* in a subset of cells. Future studies of these genes should address whether these genes also act redundantly in mammals.

## Introduction

Identifying regulators of the intermediate steps that link pluripotency and terminal differentiation is a fundamental challenge in developmental biology. These regulators are comparatively poorly understood for most tissues due to the difficulty of recognizing and isolating cells in these transient intermediate states (“progenitors”) and their complex combinatorial logic. Individual transcription factors (TFs) acting at these stages often have broad and diverse expression domains that don’t correlate well with specific tissue or cell types [1], with multiple TFs typically acting together to specify any given intermediate progenitor. Therefore, loss of function can lead to pleiotropic phenotypes, while partial redundancy between regulators can lead to reduced penetrance, making it hard to determine the relationship between expression and biological function. Large-scale screens for gene pairs with synthetic phenotypes, as has been done for yeast [2] can identify genes acting in parallel, but screening at that scale is not feasible in animals. We are overcoming these challenges with a systematic approach to define pleiotropic and redundant progenitor TFs in *Caenorhabditis elegans*, a simple model organism where lineage relationships are already understood, large-scale gene expression resources allow rapid identify patterns of TF overlap, and powerful tools exist for characterizing mutant phenotypes across all embryonic cells. Previous studies of genetic redundancy in *C*. *elegans* have prioritized gene pairs for synthetic lethality testing based on similar functional interactions [3,4], expression patterns [5] and homology or conservation [6,7].

Progenitor cells are easily identified in *C*. *elegans* because the relationship between cell lineage and fate is known and invariant[8,9]. The first several embryonic divisions give rise to founder cells, some of which have clonal or partially clonal cell fates. Most cells, however, retain a multipotent state until the final round of embryonic cell divisions, when two daughters adopt such different fates as a neuron and an epithelial tube or neuron and hypodermal (skin) cell. Thus, any TF expressed in a non-clonal progenitor cell or group of lineally related cells (i.e. lineage) at any time after the earliest cell divisions but prior to the final round could play a role in progenitor identity. Despite this potential, genetic studies have identified numerous regulators of both early founder cell identity [10–16] and of terminal fate[17–19], but fewer regulators of intermediate progenitor identity. Automated methods to track cell lineages from confocal microscopy image series have allowed quantitative expression measurements for over 200 transcription factors across every cell of *C*. *elegans* embryos [1,20–22], and this EPIC (Expression Patterns In *C* *aenorhabditis*) dataset suggests many candidate regulators of progenitor identity [1,23]. Computer-aided cell tracking of mutant embryos can confirm these regulators by identifying a wide range of pleiotropic defects, from wholesale fate transformations to subtle defects in cell migration or division timing [10,14,24–27].

Many previous studies of TF function relied on reporter gene expression to infer developmental defects. We reasoned that the complex patterns of cell cycle length asynchrony and cell migration that occur in later embryos may allow identification of defects at single cell resolution without such reporters. We used this approach to characterize the developmental role of the candidate progenitor regulator *ceh-36*, which encodes an *orthodenticle*/OTX homeodomain family transcription factor orthologous to mammalian OTX1, OTX2 and CRX proteins. A *ceh-36* reporter is expressed in multiple progenitor cells, encompassing the precursors of 248 terminal cells with diverse fates including neurons, glia, the excretory (renal) system, visceral and body muscles, epidermal and rectal epithelial cells[1]. Vertebrate OTX factors are similarly expressed and required in precursors of diverse tissues [28–37], suggesting these factors could be conserved regulators of progenitor identity. However, previous studies of *ceh-36* mutants identified defects only in the embryonic specification of four neurons [38–40]. The large number of expressing cells combined with the small number of cells known to require *ceh-36* raises the question of whether *ceh-36* is required across most expressing cells or only a minority of these cells.

We found that *ceh-36* null mutants are viable embryonically, with partially penetrant larval lethality and superficially normal morphology. Cell lineage tracing of *ceh-36(-)* embryos revealed variably penetrant defects in cell division patterns or cell migration in over 100 cells that normally express *ceh-36*. Double mutants lacking both *ceh-36* and the coexpressed PITX-family homeobox gene *unc-30* exhibited 100% synthetic lethality and severe morphological defects. These double mutants have dramatically increased rates of defective cell division and migration in coexpressing cells, indicating *ceh-36* and *unc-30* act in parallel to regulate the development of these cells. This provides the first evidence for genetic redundancy between OTX and PITX homeodomain factors, two *bicoid* class TFs that are predicted to bind similar sequences, yet diverged prior to the radiation of metazoan species.

## Results

### *ceh-36* is required for larval progression but not morphogenesis

**Fig 1:**
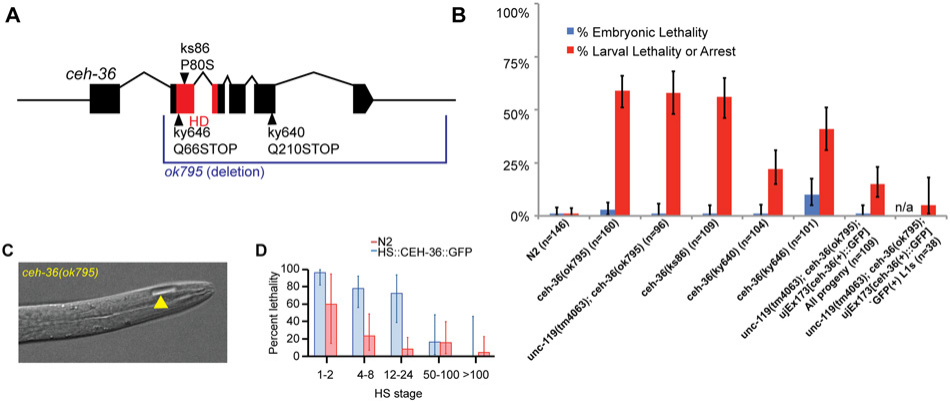
*ceh-36* mutants have partially penetrant larval lethality and mild morphological defects. A) Gene model of *ceh-36* showing alleles. B) Embryonic and larval arrest rates for *ceh-36* mutants. C) Example of a *ceh-36* mutant L1 with a head “vacuole” (arrowhead). D) Heat shock-induced embryonic lethality for embryos treated with 30 minute heat shock at 32°C at the specified stages for a strain expressing HS::CEH-36::GFP and N2 (no transgene control) (*N* for each stage in parentheses).

A *ceh-36* deletion allele that removes the majority of coding regions, including the homeodomain, was annotated as embryonic lethal in WormBase based on limited previous characterization [40,41]. After outcrossing, we found that nearly all embryos homozygous for this allele hatched, while ~60% of animals arrest as larvae (Table 1, Fig. 1A, B, S1 Table). The remainder of *ceh-36(ok795)* animals survived to adulthood and were fertile. Most arrested larvae had normal body morphology, with 5.6% of L1s containing a small bubble-like “vacuole” at the tip of the head (Table 2, Fig. 1C). Two other *ceh-36* alleles predicted to eliminate or alter the homeodomain displayed similar rates of larval arrest (Table 1), suggesting this is the null phenotype. A fourth allele, *ky640*, which truncates the protein but is predicted to encode a complete homeodomain, displayed lower lethality rates, suggesting it leads to partial loss of function. An extrachromosomal genomic fosmid transgene containing CEH-36::GFP (+) rescued *ceh-36(ok795)* larval lethality; the 85% survival in this strain corresponds to nearly 100% after accounting for the 25% rate of transgene loss (Table 1). Consistent with this, 95% of CEH-36::GFP-positive L1s survived. Ectopic expression of CEH-36::GFP under the control of a heat-shock promoter caused extensive lethality when induced prior to the 50-cell stage, while later induction had little effect (Fig. 1D), indicating that CEH-36 is toxic when expressed in these early embryonic cells, but not in later cells. We conclude that *ceh-36* is required for robust larval viability but not for gross morphology or embryonic viability.

**Table 1:** Alleles and viability phenotypes.

(Maternal) Genotype	% Embryonic Lethality	% Reaching L4 by 6 days	n
N2	1%	99%	146
*ceh-36(ok795)*	3%	41%	160
*unc-119(tm4063); ceh-36(ok795)*	1%	42%	96
*unc-119(tm4063); ceh-36(ok795); ujEx173[unc-119(+) ceh-36(+)*::*GFP](all progeny of non-unc mothers)*	1%	85%	109
*unc-119(tm4063); ceh-36(ok795); ujEx173[unc-119(+) ceh-36(+)*::*GFP](GFP(+) L1s)*	n/a	95%	38
*ceh-36(ks86)*	1%	44%	109
*ceh-36(ky640)*	1%	78%	104
*ceh-36(ky646)*	10%	59%	101
*unc-30(ok613)*	0%	100%	87
*unc-30(ok613); ceh-36(ok795)*	54%	0%	56
*unc-30(ok613); ceh-36(ok795); ujEx173[unc-119(+) ceh-36(+)*::*GFP](GFP(+) embryos)*	0%	75%	133

**Table 2:** Reporter defects in ceh-36.

Reporter (Cells assayed)	Genotype	% Defective	n
GCY-5::GFP (ASE)	Wildtype	0%	58
	*ceh-36(ok795)*	12%	57
SAMS-5::GFP (MI)	Wildtype	0%	51
	*ceh-36(ok795)*	100%	54
HLH-6::GFP (Pharyngeal glands)	Wildtype	0%	50
	*ceh-36(ok795)*	29%	68
FKH-4::GFP (Left intestinal muscle)	Wildtype	0%	56
	*ceh-36(ok795)*	14%	65
FLP-1::GFP (AVK)	Wildtype	0%	52
	*ceh-36(ok795)*	2%	51

### *ceh-36* is expressed in bilateral lineages that produce diverse tissues

**Fig 2:**
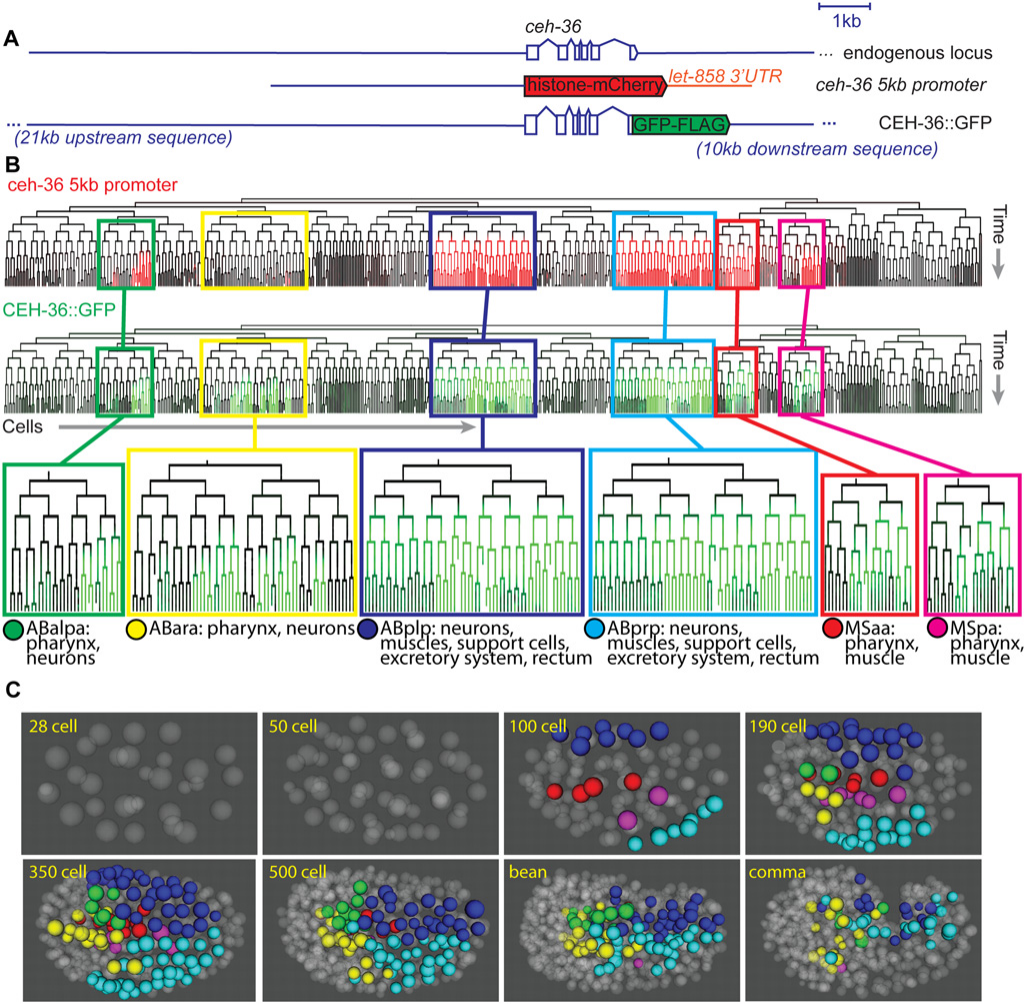
CEH-36 protein reporter and endogenous message are expressed dynamically in ABpxp and other lineages. A. Schematic of *ceh-36* reporter constructs. B. Expression of *ceh-36* promoter::histone-mCherry reporter (top, red) and CEH-36::GFP rescuing translational reporter (green, bottom) in each cell of the lineage. C. Positions of CEH-36::GFP expressing cells over time, shown in 3D projection with expressing nuclei color coded by lineage of origin as outlined at the bottom of panel B.

We previously analyzed expression of a 5-kb *ceh-36* promoter fusion reporter and identified expression in several major lineages (Fig. 2, S1 Fig) [1]. Since this reporter may not contain all relevant regulatory sequences, we generated transgenic strains using a fosmid clone from the “Transgeneome” project [22] where CEH-36 protein is fused to GFP in the context of the endogenous locus (Fig. 2A). This transgene rescues the higher-penetrance *ceh-36* mutant lineage defects and larval arrest phenotype described below (Table 1). Using lineage analysis, we identified all CEH-36::GFP expressing cells through the comma stage, at which point the embryo starts to move. CEH-36::GFP is expressed in progenitors of 248 terminal cells from six lineages that together produce a mix of diverse cell types including pharyngeal cells, muscles, neurons, glia and specialized cell types, and programmed cell deaths (Fig. 2B, C). CEH-36::GFP is predominantly (>90%) expressed symmetrically between left and right symmetric lineages, despite left-right asymmetric expression and function for two of the four neurons previously shown to require *ceh-36* [38,40]. The spatial expression pattern is similar to the previously analyzed *ceh-36* promoter fusion (Fig. 2B, S2 Fig), but includes additional expression in the ABara lineage. We also analyzed a previously published 2-kb promoter fusion reporter [38,40,42] that we found is expressed in the MSa, MSp and ABalpa lineages but not ABara, ABplp or ABprp, indicating the existence of multiple regulatory elements for *ceh-36* in different lineages (S3 Fig).

The CEH-36 protein fusion reporter exhibits complex dynamics that we confirmed by single molecule RNA-FISH (smFISH) [43] of endogenous *ceh-36* mRNA (see below). Expression in the ABpxp, MSaa, and MSpa lineages begins between the 50-cell and 100-cell stages and decreases in most cells after 2–3 cell cycles, prior to morphogenesis (Fig. 2B). However, a few cells maintain stable expression much longer, up to at least comma stage. The CEH-36::GFP expressing cells include progenitors of three neurons previously shown to require *ceh-36* (MI, AWCL and AWCR), with additional stronger expression in AWCL, AWCR and the fourth *ceh-36*-requiring cell, ASEL, beginning after the worm begins to elongate and twitch [38–40]. In total, we found expression of *ceh-36* in progenitors of over 30% of embryonic cells suggesting it could play a broad role in embryonic patterning. Its early and transient expression in progenitor cells suggested that *ceh-36* might be an important regulator of progenitor identity or function.

### Quantitative analysis identifies broad roles for *ceh-36* in regulating cell cycle timing and cell position

**Fig 3:**
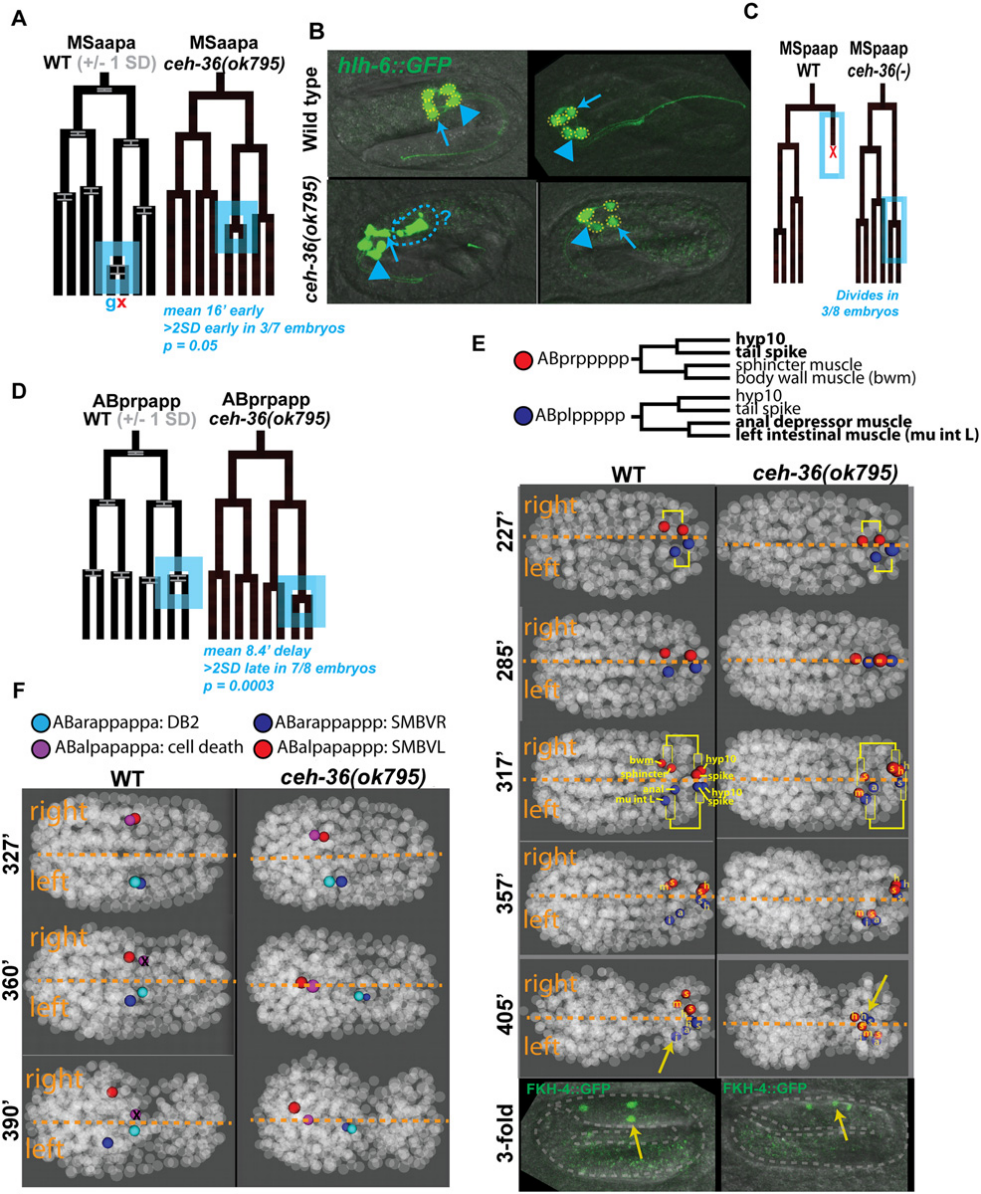
Examples of cell lineage and position defects observed in *ceh-36* mutants. A) Early division of the MSaapapa cell (blue box). Cell fates under the WT tree labeled as **g =** pharyngeal gland, **x** = programmed cell death. Error bars in WT tree represent standard deviation of division time. Data for mutants are from a representative defective embryo. B) Morphological defects in pharyngeal glands (marked with *hlh-6*::GFP) in *ceh-36(ok795);* anterior gland cells (arrowhead) and posterior gland cells (arrows) show defects in organization (left panel, disorganized morphology and possible extra cells) and number (right panel, one missing posterior gland cell, yellow dashed circles) in *ceh-36* mutants C) Failed cell death of MSpaapp, which instead divides. D) Late division of ABprpapppp (blue box), which produces AIMR and CEPVR neurons. E) Left-right migration defects in ABplppppp and ABprppppp lineages (cells with migration defects in bold). Sister cells denoted with yellow lineage brackets. FKH-4::GFP marks mu int L (yellow arrow), mu int R and anal depressor (S4 Fig). F) Anterior-posterior migration defects of SMBV cells and their sisters (DB2 and cell death). ABalpapappa fails to die in 7/8 *ceh-36(ok795)* embryos.

**Fig 4:**
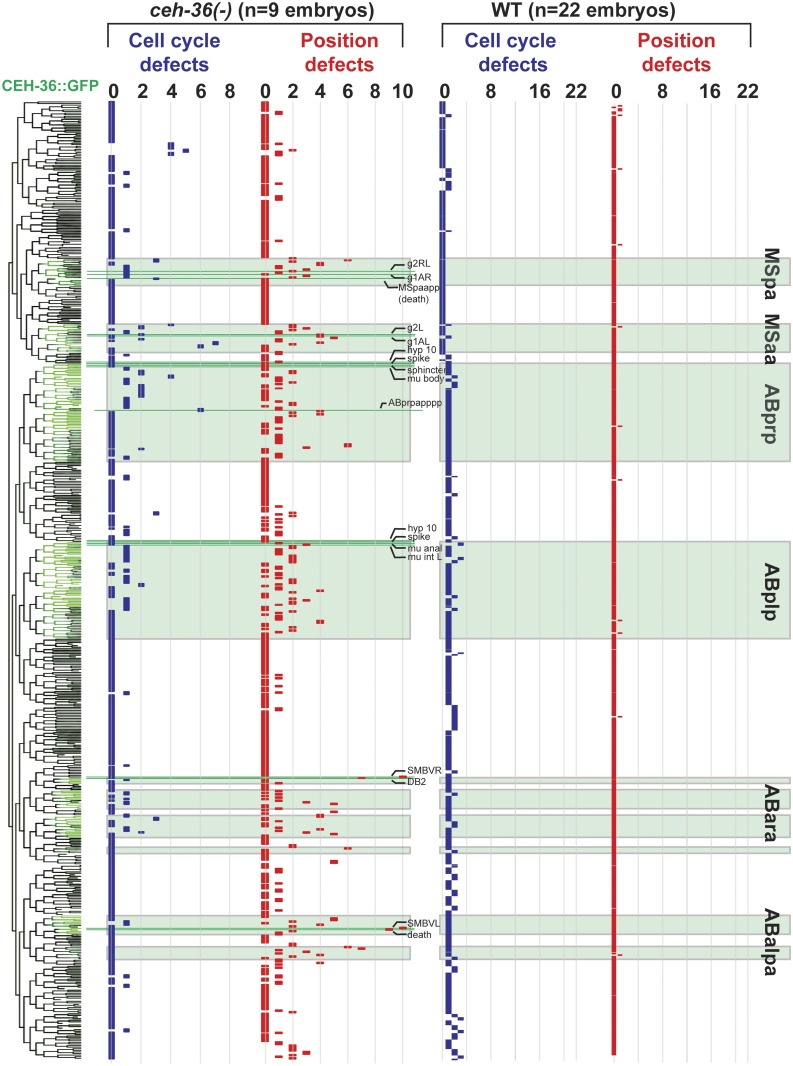
Lineage distribution and penetrance of cellular phenotypes in *ceh-36* mutants. Lineage diagram with CEH-36::GFP expression (green) for reference. Columns show frequency of cell cycle (blue) and position (red) defects for each terminal cell across nine *ceh-36* mutant embryos (eight carrying the *ok795* deletion allele and one carrying *ky646*). CEH-36::GFP expressing lineages are labeled with green boxes. Lineage positions of cells discussed in the text or in Fig. 3 are labeled (pharyngeal glands = g1, g2, others labeled as described). Rare defects in progenitor cells were added to defects in terminal cells, allowing a few cells to have more than 9 cumulative defects.

The lack of obvious morphological defects in *ceh-36* mutants suggests that *ceh-36* might play a minimal role in the development of most expressing cells. To test this, we searched for defects in lineage patterns and cell migrations in mutant embryos using automated cell tracking. We examined quantitative features of embryonic development, including timing and patterns of cell division, division orientation, and positions in eight *ceh-36(ok795)* embryos through the comma stage (~400 minutes after fertilization, when nearly all cell divisions have occurred) and compared these phenotypes to a wild-type reference set [27] (see Materials and Methods) and to three embryos expressing a rescuing CEH-36::GFP transgene. We also examined one embryo carrying a second predicted *ceh-36* null mutation (*ky646*). As detailed below, we found that many cells in *ceh-36(-)* embryos have partially penetrant defects in both cell cycle timing and cell position (Figs. 3,4, S2 Table). In total, 5.1% (495/9636) of cells in *ceh-36(ok795)* embryos were defective in cell division or position, compared with 0.3% (85/26171) of cells in wild-type control embryos (p < 10^-220^; chi-squared test). This suggests that *ceh-36* is broadly important for robust development across its expressing cells.

### Loss of *ceh-36* disrupts robust control of cell division and cell death

The *C*. *elegans* lineage is composed of an invariant pattern of cell divisions and deaths. In wild-type embryos, the division timing is highly stereotyped, with most cells having variability in cell cycle length of less than 5% [27] [44]. We identified 49 cells with cell cycle or lineage timing defects in at least one *ceh-36* mutant embryo (Figs. 3A, D, 4, S2 Table), defined as cells dividing both three standard deviations and at least five minutes earlier or later than expected, not dividing at all, or dividing inappropriately. In addition, three cells failed to undergo programmed cell death when expected, as recognized by the characteristic pattern of chromatin compaction observed for histone-mCherry. For example, in three embryos, MSpaapp, which normally is the first embryonic cell to undergo apoptosis, instead survived and divided, with both sisters migrating into the pharynx to adopt unknown fates (Fig. 3C). In some cases, cells not passing our threshold for defect calling appeared to have different mean cell cycles or positions. For example 35 of 49 cells with cell cycle defects in one or more embryos also had a nominally significant difference in mean cell cycles (p < 0.1; FDR < 0.15; S2 Table). The CEH-36::GFP fusion protein is expressed in precursors of 86%(12/14) of cells with cell division timing defects in two or more *ceh-36(-)* embryos, and 60% (21/35) of cells with defects in one embryo. This is significantly more than the 30% of all cells that express CEH-36::GFP (chi squared p < 2 × 10^-6^). CEH-36::GFP is also expressed in all of the cells with supernumerary divisions or failed cell death.

### *ceh-36* is required for cell migration and bilateral symmetry

Cell positions are also highly consistent between wild-type embryos, allowing us to identify cell migration defects by comparing cell positions between *ceh-36* mutant and wild-type embryos. We identified 124 cells whose deviation from expected position was at least 3.5 standard deviations greater than in the wild-type set and that had aberrant neighbors as defined by an empirical neighbor-distance score (S2 Table; see Methods). Position defects were strongly enriched in expressing cells; 81% (55/68) of cells with position defects in two or more embryos normally express CEH-36::GFP. By comparison, in 22 wild-type embryos examined, only 13 cells had defective positions, in one embryo each.

A cell could be misplaced because of a defective migration, in which case it would have both different position and different neighbors than in the wild type. Alternatively, a cell could be misplaced because its normal position is occupied by another cell that migrated inappropriately, in which case its position relative to its normal neighbors would be unchanged. We used these criteria to classify 50 cells with position defects by examining their position and neighbors in 3D visualizations (Fig. 3E, F). We scored 82% (41/50) of cells as likely defective migrations, while 9/50 (18%) defects could be explained by defective migration of other cells (S3 Table). 100% (18/18) of higher-penetrance (seen in at least three of eight *ok795* embryos) position defects examined were scored as likely migration defects. The migration defects include both cells that undergo novel migrations in the mutant (Fig. 3E) as well as cells that fail to undergo their expected migrations (Fig. 3F). The cells scored as possible secondary defects were less penetrant, with each identified as defective in one or two embryos. Still, most low-penetrance defects (23/32) were scored as likely migration defects

We observed dramatic defects in eight laterally positioned cells that were born in the correct position but subsequently migrated across the midline to the opposite lateral side of the embryo, sometimes displacing the position of their bilateral counterpart (e.g. Fig. 3E). These lateral migration defects occurred on both sides of the embryo (3 L→R, 5 R→L) and include diverse cell types: neurons (I1R and I2L), pharyngeal cells (pm3R and mc1DR), rectal cells (left intestinal muscle and anal depressor muscle), and tail cells (Hyp10 and tail spike). These defects were all low penetrance (seen in one or two of eight *ok795* embryos), but we saw no defects of this class in the 22 wild-type control embryos, and all eight of these cells normally express *ceh-36*. This indicates that *C*. *elegans* cells’ lateral position is not merely a result of their birth position but is regulated by factors that include *ceh-36*.

We determined that lateralization defects are maintained through embryonic elongation and not corrected by subsequent cell movements by examining worms expressing FKH-4::GFP, a marker of three visceral muscles (left and right intestinal muscles and anal depressor; Fig. 3E, S4 Fig). 100% of both wild-type and *ceh-36* mutant elongated (pretzel-stage) embryos have three FKH-4(+) cells, indicating that *ceh-36* is not necessary for FKH-4 expression. However, one FKH-4(+) cell is laterally mispositioned in 14% of *ceh-36(-)* embryos (Table 3; Fig. 3E). This is consistent with the left-right migration phenotype and low penetrance observed in our lineage data (1/8 *ok795* embryos, 12.5%), increasing confidence in the low-penetrance defects identified by lineage analysis.

**Table 3:** Morphology phenotype frequencies.

Genotype	Fluid in head	Vab	Nob	Fluid from excretory system	n
Wildtype	0%	0%	0%	0%	52
*ceh-36(ok795)*	6%	3%	0%	0%	72
*unc-30(ok613)*	0%	0%	0%	0%	51
*unc-30(ok613); ceh-36(ok795)*	0%	47%	57%	21%	53
*unc-30(ok613); ceh-36(ok795); Ex unc-119(+) ceh-36(+)*	0%	0%	0%	0%	70

Includes unhatched embryos.

### Pharyngeal gland defects correlate with *ceh-36(-)* larval lethality

Multiple pharyngeal gland cell precursors had cell cycle and position defects in *ceh-36* mutants. For example, the daughters of the MSaapapa cell normally produce a pharyngeal gland cell and a programmed cell death and the early division of this cell was the largest division-timing defect we observed in *ceh-36* mutants (Fig. 3A). Precursors of four of the five pharyngeal gland cells express CEH-36::GFP and all four of these had partial penetrance defects in cell cycle or position (Figs. 3A, 4). Since pharyngeal gland cells are known to be required for feeding and viability [45], we examined them for additional defects by examining expression of the pharyngeal gland marker *hlh-6*::GFP in elongated *ceh-36(ok795)* embryos. We observed altered pharyngeal gland morphology in 20% of *ceh-36(ok795)* elongated embryos. An additional 9% of embryos were missing one or more *hlh-6*::GFP-positive cells (Fig. 3B, Table 2), suggesting that *ceh-36* regulates not only gland cell cycle patterns and morphology but also terminal fate. While only 41% (23/56) of larvae with normal gland morphology arrested prior to the L4 stage, 92% (46/50) of larvae with abnormal gland morphology arrested. Thus, defects in pharyngeal gland morphology predict larval arrest in *ceh-36* mutants.

### *ceh-36* mutant defects are partially penetrant

Defects occurred in 223 unique cells, typically with low penetrance; only 82/223 (37%) cells were defective in two or more (of eight) *ok795* embryos. Most of the defective cells normally express CEH-36::GFP (77%), significantly more than the 30% fraction of all cells that express *ceh-36* (p < 10^-90^, chi-squared test). Most of the defective cells that do not normally express *ceh-36* were only called as defective in one embryo. Still, even defects seen in a single embryo were enriched in expressing cells (59% of such cells express CEH-36::GFP). While cells with prior cell cycle defects were 2.9-fold more likely to have position defects (p < 10^-9^), 90% of cells with position defects had no detectable cell cycle defect. Only 22 expressing cells had defects in at least 50% of analyzed embryos (e.g. Fig. 4, S2 Table). The low penetrance of most individual defects may explain the viability of *ceh-36(-)* embryos.

We determined whether cells with low penetrance defects have noticeable defective terminal positions or numbers by examining several fluorescent markers expressed in these cells (Table 2). Reporters for two cells previously reported as requiring *ceh-36* (MI(*sams-5*) and ASEL(*gcy-5*)) showed the expected terminal defect frequencies in the *ceh-36* deletion. As described above, the visceral muscle reporter FKH-4::GFP and the pharyngeal gland reporter *hlh-6p*::GFP also showed terminal position defect frequencies consistent with the observed embryonic defects. Finally, a FLP-1::GFP reporter reported as expressed in the AVK neuron (which expresses *ceh-36* but was not identified as defective in our analysis) showed little or no terminal defects (~2%). Given that the mutant embryos hatch without major morphological defects despite an average 40 cells with position defects and 10 cells with altered division timing, development of *C*. *elegans* embryos must be robust to a substantial amount of developmental error.

### *ceh-36* mutant defects are clustered in related cells

The rescuing CEH-36::GFP transgene expression is typically strongest several divisions before the birth of the terminal cells where most defects were identified, suggesting that defects in *ceh-36(-)* may result from regulatory events occurring in mitotic progenitor cells. If this is true, partially penetrant defects should preferentially co-occur in closely related cells within a given embryo. We identified 71 examples of defective sister cell pairs in *ceh-36*-expressing cells. We found preferential co-occurrence of defects in sisters for seven embryos (p < 0.001) by using a bootstrap evaluation, and this co-occurrence was only significant in cells expressing *ceh-36*. This along with the early and dynamic CEH-36::GFP expression suggests that *ceh-36* regulates development in part through its activity in progenitor cells, rather than the terminal cells that exhibit the defects.

### Lineage defects are observed with multiple *ceh-36* alleles and are rescued by CEH-36::GFP

To confirm that most defects identified in *ceh-36(ok795)* embryos result from loss of *ceh-36*, we specifically examined high-penetrance (≥6 of 8 *ok795* embryos) position defects in an embryo carrying a second predicted *ceh-36* null mutation (*ky646*). We found four of the five cells examined had similar defects in this embryo. We examined these cells in two *ceh-36(ok795)* embryos expressing CEH-36::GFP, and one embryo with mosaic CEH-36::GFP expression, and found that these defects were rescued in all CEH-36::GFP expressing cells. Taken together, these results show that *ceh-36* regulates the robustness of cell cycle and migration patterns in many cells. Our analysis did not explicitly test for changes in cell fate, but given the known role of *ceh-36* in fate specification [38–40], there may be additional unidentified cells with defects in fate, but not position or cell cycle timing.

### Expression of *ceh-36* is distinct from its *OTX* paralogs *ceh-37* and *ttx-1* but overlaps substantially with *unc-30/PITX* in early embryos

**Fig 5:**
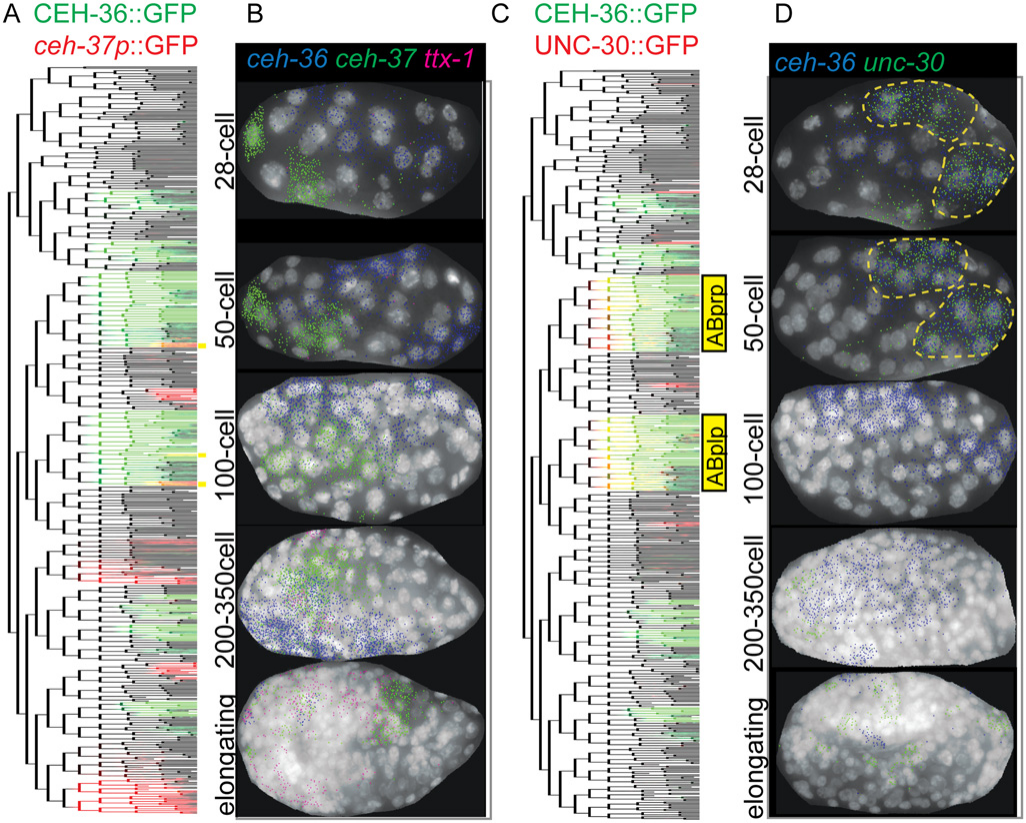
Embryonic coexpression patterns of *C*. *elegans* OTX and PITX factors. A, B) Expression overlap of (A) CEH-36::GFP and *ceh-*37 promoter::GFP or (C) CEH-36::GFP and UNC-30::GFP mapped onto a reference lineage [21]. Cells with overlapping expression are SAAV(L/R), AWC(L/R), DB1, DB3, RMEV and the excretory duct, pore and canal cells. RMEV and the canal cell have sustained *ceh-36* expression joined later by *ceh-37*, while all others express first the *ceh-36* reporter, then the *ceh-37* reporter, with minimal temporal overlap. B, D) Coexpression of RNA as identified by single molecule RNA-FISH [43] at various stages for (C) *ceh-36*, *ceh-37* and *ttx-1* or (D) *ceh*-36 and *unc-30* in embryos at the indicated stages.

Most defects in *ceh-36(ok795)* have low penetrance, so other transcriptional regulators likely function in parallel with *ceh-36* to ensure robust development. Therefore, we searched for transcription factors that might act redundantly with *ceh-36* (Fig. 5). Previous work demonstrated that the three *OTX* family members *ceh-36*, *ceh-37*, and *ttx-1* can rescue the others’ mutant phenotypes when expressed in the appropriate cells [39]. We asked if these genes’ early embryonic expression overlaps with that of *ceh-36* by lineage analysis of fluorescent reporters and single molecule (sm)RNA-FISH [43]. Lineage analysis of a *ceh-*37 promoter-fusion reporter[46] identified ten cells where its expression overlaps spatially but not temporally with *ceh-36*; the *ceh-37* reporter is expressed after CEH-36::GFP in these cells (Fig. 5A). The *ceh-37* reporter is also expressed in several lineages that do not express CEH-36::GFP. *ceh-37* transcripts identified by smRNA-FISH did not overlap with positions of *ceh-36* transcripts prior to the 50-cell stage and there was only a small amount of overlap between the 50 and 200 cell stages (Fig. 5B, S5 Fig). We could detect no embryonic expression of a *ttx-1* promoter reporter prior to morphogenesis and little or no overlap between *ttx-1* and *ceh-36* transcripts by smRNA-FISH (Fig. 5B). We examined these genes’ expression in *ceh-36(ok795)* by smRNA-FISH and observed no *ceh-36* transcripts and no changes in *ceh-37* or *ttx-1* expression. We also observed no substantial increase in *ceh-36(ok795)* lethality after *ttx-1* or *ceh-37* RNAi. This indicates that most *ceh-36*-expressing cells do not express other *OTX* homologs in wild-type or *ceh-36* mutant embryos, and redundancy with these factors is unlikely to explain the low penetrance of most *ceh-36* mutant defects.

We mined the EPIC database of embryonic expression patterns [1,23] for additional factors coexpressed with *ceh-36*, and identified substantial coexpression with the PITX homolog *unc-30*. An UNC-30::GFP fosmid “Transgeneome” reporter [22] was transiently expressed at the same time as CEH-36::GFP in the descendants of the ABplp and ABprp progenitor cells (together “ABpxp”), which give rise to diverse cell types, but not in other CEH-36-expressing lineages. We confirmed this expression overlap between endogenous *ceh-36* and *unc-30* transcripts in ABpxp-derived cells by lineage tracing (Fig. 5C) and observed significant overlap of these genes’ endogenous transcripts by smRNA-FISH between the 28-cell and 50-cell stages (Fig. 5D). *ceh-36/OTX* and *unc-30*/*PITX* both encode bicoid-type homeodomains that are predicted to bind similar target sequences [47,48]. In addition, the combined frequency of position and cell cycle defects in *ceh-36(ok795)* embryos was lower (3%) in the ABpxp lineages than in other CEH-36::GFP-expressing cells (12%) suggesting that *ceh-36* may have more redundancy in ABpxp than in other lineages. This suggested the possibility that these two factors might act redundantly to regulate the development of the ABpxp lineages.

### *unc-30* and *ceh-36* are redundantly required for embryonic and larval viability

In addition to its early ABpxp expression, we observed UNC-30::GFP in the six embryonic type D GABA-ergic motorneurons as well as a few other neurons (PVP, AWA, ASG, AIB, ASI and GLR) at morphogenesis (bean stage), consistent with the known role of *unc-30* in the terminal differentiation of type D neurons [49] (S6 Fig). Consistent with the phenotypes of other *unc-30* alleles, the deletion allele *unc-30(ok613)* is uncoordinated yet fully viable, with no embryonic or larval arrest (Table 1).

We tested for redundancy between *unc-30* and *ceh-36* by examining the progeny of a strain homozygous for both *unc-30(ok613); ceh-36(ok795)* and carrying the rescuing extrachromosomal CEH-36::GFP fosmid. Animals that had lost the rescuing transgene displayed 100% lethality (54% embryonic, 46% larval), while embryos expressing CEH-36::GFP had no embryonic lethality and low larval arrest rates (Table 1), with 75% progressing to L4. The residual larval arrest rate could result from transgene mosaicism or incomplete rescue by the CEH-36::GFP transgene. This indicates that *ceh-36* and *unc-30* are redundantly required for viability.

**Fig 6:**
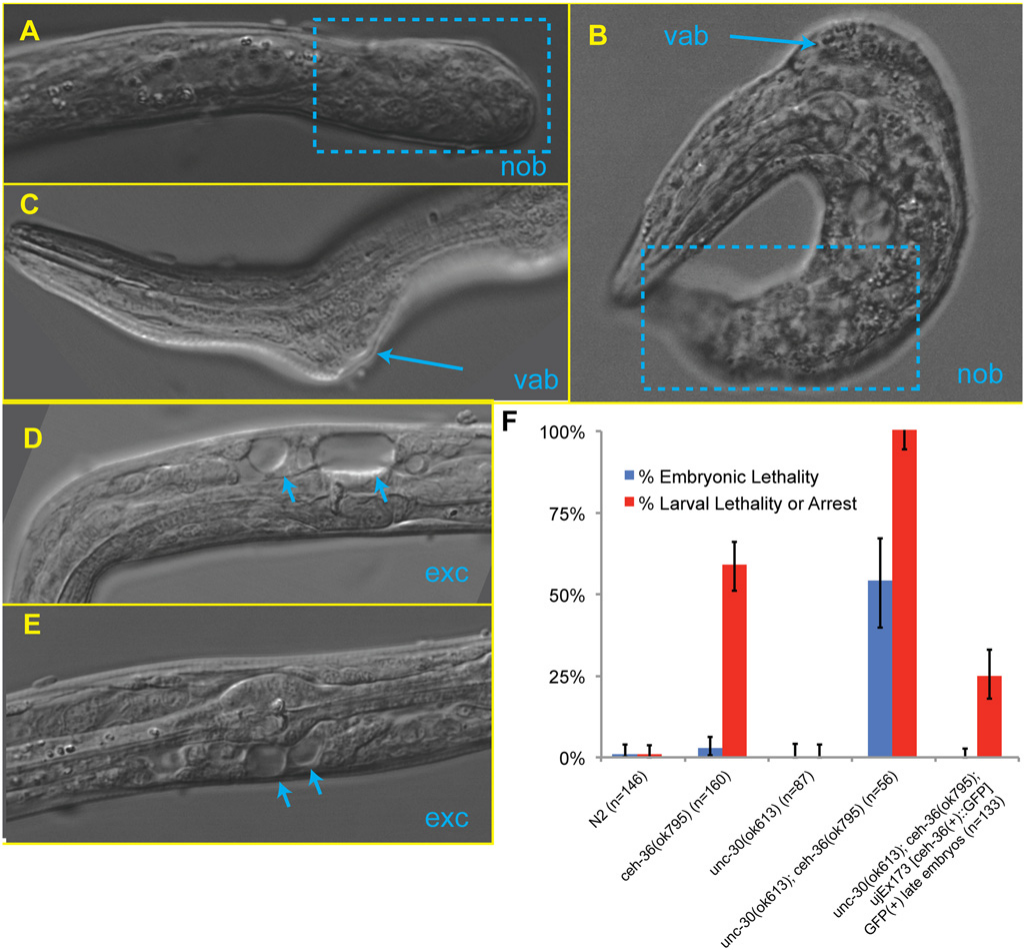
Examples of phenotypes in *unc-30;ceh-36* double mutants. A, B) Failure of posterior morphogenesis resulting in a “no-backend” (Nob) phenotype (boxed). B, C) Variable abnormal defect (Vab) in body morphology (arrows). D-E) “Bubble” phenotypes characteristic of a blockage (Exc) in the excretory system (arrows). F) Embryonic and larval arrest rates for *unc-30* and *ceh-36* single and double mutants.

The *unc-30(ok613); ceh-36(ok795)* double mutants displayed visible phenotypes characteristic of defects in ABpxp-derived cells not observed in either single mutant (Table 3, Fig. 6). These included variable abnormalities in body morphology (Vab) defects, which are also seen when ABpxp-derived cells fail to act as a substrate for hypodermal enclosure [50,51], “no backend” (Nob) tail defects characteristic of severe defects in patterning posterior cells including many derived from ABpxp [52], and a “rod-like” arrest posture and large edemas near the pharynx characteristic of defects in the excretory system [53], which is formed by descendants of ABpxp cells. Double mutants did not contain the more anterior head “vacuoles” we saw in *ceh-36(ok795)* single mutants; however this phenotype could be masked by the more severe Vab and excretory phenotypes. Taken together, *ceh-36* and *unc-30* are redundantly required for viability and for aspects of normal development associated with cells produced by ABpxp.

### *ceh-36* and *unc-30* co-regulate lineage patterning in ABpxp

**Fig 7:**
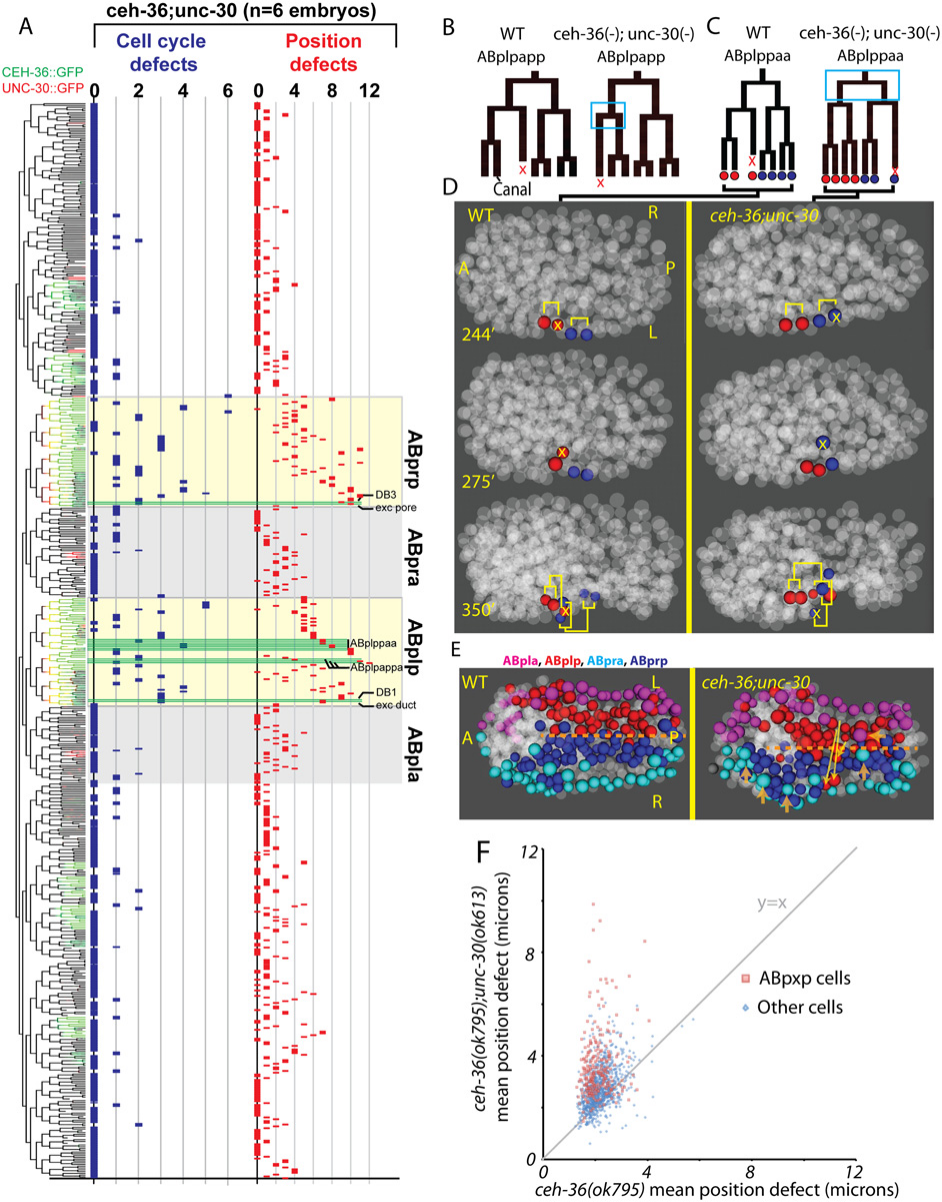
unc-30;ceh-36 double mutant lineage phenotypes. A) Frequency of defects in each cell of *unc-30;ceh-36* double mutants (compare to Fig. 4). Expression tree shows CEH-36::GFP (Green) and UNC-30::GFP (Red). B-D) Examples of “lineage reversals” where the division patterns of daughters of ABplpappa (B) or ABplppaa (C) reverse their patterns of division and death and migration (D). E) Positions of ABpxp (express *ceh-36* and *unc-*30) and ABpxa (express neither *ceh-36* nor *unc-30*) derived cells in the wild type model and a *unc-30;ceh-36* double mutant embryo showing ABpxp-derived cells that have crossed the midline (yellow arrows show deviation from wild-type mean position) and incomplete migration of ventral epidermal cells (derived from ABpxa) (orange arrows). F) Mean magnitude of position defects in *ceh-36* and double mutants.

The highly penetrant viability and morphological phenotypes of *unc-30(ok613); ceh-36(ok795)* double mutants led us to hypothesize that these animals would have more frequent cell lineage and position defects in the cells that normally coexpress both factors. We tested this by automated lineage analysis of six *unc-30(ok613); ceh-36(ok795)* embryos that had lost the rescuing CEH-36::GFP transgene (Fig. 7, S2 Table). We observed a significant increase in cell cycle and cell position defects in the ABpxp lineages of *unc-30; ceh-36* double mutants as compared to *ceh-36* alone. Double mutant embryos averaged 15.5 cell cycle defects and 94.7 position defects per embryo in ABpxp compared with 2.25 and 11 in *ceh-36* single mutants. We also saw a smaller increase in position defects for cells that do not normally express either CEH-36::GFP or UNC-30::GFP (64 vs 26.1), consistent for a role of the ABpxp cells in migration of cells from other lineages. In contrast, we saw no corresponding increase in cell cycle defects in double mutants for nonexpressing lineages (Fig. 7), and no corresponding defects in UNC-30 single mutants (S7 Fig).

We observed an increased co-occurrence of defects in sister cell pairs in the ABpxp lineages of double mutants (27.2 sisters pairs per embryo) compared with *ceh-36(ok795)* alone (3.8 sister pairs per embryo). Because *unc-30* expression in the ABpxp lineage is even more transient than that of *ceh-36*, the increased co-occurrence of defective sister cells likely results from primary defects in progenitor cells. We individually examined *ceh-36(ok795)* single mutant and *unc-30(ok613); ceh-36(ok795)* embryos for “defect trios” of a defective mother with two defective daughter cells. While *ceh-36(ok795)* embryos have on average 1.9 of these defect trios (none in ABpxp), double mutant embryos average 13.5 defect trios (10.8 in ABpxp). We observed only one defect trio in three *unc-30(ok613); ceh-36(ok795)* embryos expressing rescuing CEH-36::GFP, indicating that these defects do not result from *unc-30(ok613)*. Together this suggests that *unc-30* and *ceh-36* cooperate to regulate robust lineage patterning of ABpxp-derived progenitor cells.

Two cell divisions (ABplpappa and ABplppaa) each displayed a novel type of defect we term “anterior-posterior reversals” in 2 of 6 double mutant embryos. In this class of defect, the anterior daughter of a division adopts the division pattern of the posterior daughter and vice versa. This was evident in the patterns of asymmetric division timing, cell death and migration (Fig. 7B-D). For example, ABplpappap, which undergoes cell death in the wild type, survives and divides in the double mutant. Meanwhile, that cell’s anterior sister ABplpappaa, which should generate RMEV and the excretory canal cell, instead undergoes programmed cell death. Consistent with this being a fate reversal, the division occurs with normal orientation and the daughters of the cell that should have died go on to adopt positions characteristic of RMEV and the excretory canal cell. Defects in these fate-reversed cells or their failure to function correctly in their new location could explain some of the excretory system edema observed in the double mutants.

We identified numerous defects in the organization of the ventral midline in the double mutants. Several ABpxp-derived cells failed to respect the midline and crossed to the opposite side; these were distinct from those seen in *ceh-36* single mutants (Fig. 7E). Also, in contrast with *ceh-36* single mutants, the double mutants had a much larger number of (presumably nonautonomous) defects in cells that normally express neither *ceh-36* nor *unc-30*. Most of these defects (43/65) were in cells derived from the ABpxa lineages in cells that should form the ventral epidermis. Previous work showed that in the process of ventral enclosure, the epidermal cells migrate over ABpxp-derived substrate cells, some of which are mispositioned in the double mutants. The “leading cells” hyp6/ABpxaappap and hyp7/ABpxaappaa, which initiate ventral enclosure, along with adjacent migrating epidermal cells hyp4, G2, and W, had the largest magnitude defects in cell position of nonexpressing cells (Fig. 7E). This suggests that *ceh-36* and *unc-30* regulate development of the ABpxp-derived substrate for normal epidermal migration and morphogenesis.

### *ceh-36* and *unc-30* regulate ABpxp-specific expression of *mls-2* and excretory system development

**Fig 8:**
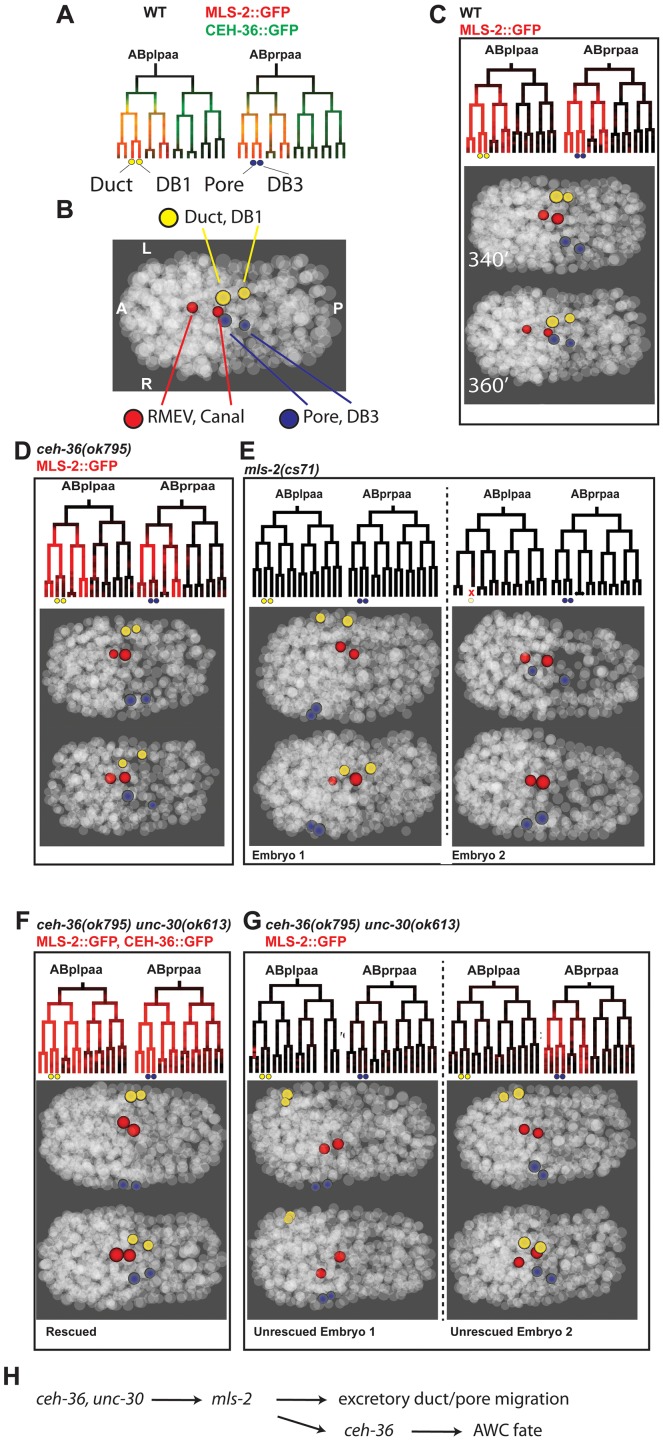
*ceh-36* and *unc-30* regulate *mls-2* and excretory system coalescence. A) Wild-type expression of CEH-36::GFP (green) precedes MLS-2::GFP (red) in the ABplpaa lineage, which produces the excretory duct and its posterior sister DB1 (yellow circles) and the excretory pore and its posterior sister DB3 (blue circles). B) Wild-type position of excretory system cells and their sisters at 360 minutes (20°C). C) Wild-type expression of MLS-2::GFP and migration patterns of excretory system cells. D) Expression of MLS-2::GFP and excretory migration in *ceh-36(ok795)* single mutants. E) Examples of defect in excretory pore and DB3 migration (left) or death of the excretory duct/DB1 parent (right) in *mls-2(cs71)* mutants. F) Expression of MLS-2::GFP and normal duct/pore migration in *unc-30;ceh-36* double mutants carrying rescuing CEH-36::GFP. G) Partial penetrance loss of MLS-2::GFP expression and failure of duct/pore migration in unrescued *unc-30;ceh-36* double mutants. Left embryo shows example of complete loss of MLS-2::GFP expression and migration failures in both duct and pore. Right embryo shows loss ot MLS-2::GFP in duct lineage but not pore lineage, pore migrates normally, while duct migration is delayed and shifted slightly anterior. H) Recursive use for *ceh-36* in AWC development.

Several ABpxp-derived cells had position defects of much larger magnitude than we observed in *ceh-36*(-) alone (Fig. 7F). Among the cells with the largest defects (average 8.9 micron (>2 cell diameters) deviation from expected position compared with 1.9 microns in *ceh-36* alone and 1.6 microns in wild type) were two sister pairs that normally produce two DB neurons and the excretory duct and G1 pore cells (Fig. 8A, B). In wild-type embryos, these cells migrate from anterior lateral positions to the ventral midline where the duct and pore cells connect with the excretory canal cell to form a continuous three-celled tube (Fig. 8C) [54].

Previous work showed that the *HMX* homeodomain transcription factor *mls-2* is required for robust development of the excretory duct and pore [55]. *MLS-2*::*GFP* is expressed in several lineages including the precursors of the excretory duct and pore (Fig. 8A, C). To determine if loss of *mls-2* leads to cell migration defects similar to those seen in *unc-30;ceh-36* double mutants, we traced the lineages and cell positions of the excretory system cells in 23 *mls-2* mutant embryos (Fig. 8D). We found migration failure or inappropriate migration into the head in 43% (10/23) of duct cells and 57% (13/23) of pore cells indicating that *mls-2* is required for robust migration of these cells. Consistent with this, previous work [55] found that 5 of 25 *mls-2* mutant larvae were missing an excretory tube cell; the difference in rates suggests that in some cases the misplaced cells may eventually migrate to the correct position, or that another cell may sometimes adopt a duct or pore fate.

We tested whether *mls-2* expression depends on *ceh-36* and *unc-30* by measuring the expression of a genomically integrated rescuing MLS-2::GFP reporter [55] in *unc-30; ceh-36* double mutant embryos. MLS-2::GFP was expressed normally in *ceh-36(ok795)* single mutant embryos (n = 6). Since MLS-2::GFP is expressed later and ~10-fold more strongly than CEH-36::GFP in the excretory duct and pore lineages (Fig. 8A), we were able to compare MLS-2::GFP expression between double-mutant embryos carrying the rescuing CEH-36::GFP with unrescued embryos. We found that in all (7/7) embryos carrying CEH-36::GFP, MLS-2::GFP was robustly expressed in both the duct and pore precursors, and the duct and pore cells migrated normally. In contrast in six embryos (12 duct/pore lineages) that had lost the rescuing transgene and expressed no CEH-36::GFP, 58% of duct/pore lineages (7/12) had no MLS-2::GFP expression, with the remaining lineages expressing MLS-2::GFP at lower levels than in wild-type or rescued embryos. Absence of MLS-2::GFP expression predicted migration defects; all seven duct or pore cells with no MLS-2::GFP expression had severe migration defects, while three of five MLS-2::GFP-expressing duct/pore cells migrated normally, sometimes with moderate delays. MLS-2::GFP expression in other lineages that don’t normally express *ceh-36* or *unc-30* was unaffected.

Discontinuities in the excretory tube are associated with formation of edemas and eventual lethality with a characteristic rod-like posture [53–56]. Thus the edemas (Table 3) and rod-like lethality we see in *unc-30; ceh-36* double mutants could be explained by the duct and pore migration defects or defects in specification of these cells or the canal cell. We conclude that *ceh-36* and *unc-30* are required for robust *mls-2* expression in ABpxp descendants that give rise to the excretory system, and that misregulation of *mls-2* may account for the observed phenotypes in those cells.

## Discussion

Our analysis of *ceh-36* and *unc-30* function across all embryonic cells highlights the complex biology of transcriptional regulation during development that would not have been discovered using traditional approaches. We showed these factors regulate distinct processes including the cell cycle, lineage patterning, cell position, and cell fate specification in many embryonic cells that go on to adopt diverse fates. These factors likely function together to regulate progenitor identity in the ABpxp lineage and *ceh-36* likely works with other unknown factors in progenitors from other lineages.

### Combinatorial lineage-specific regulatory networks

**Fig 9:**
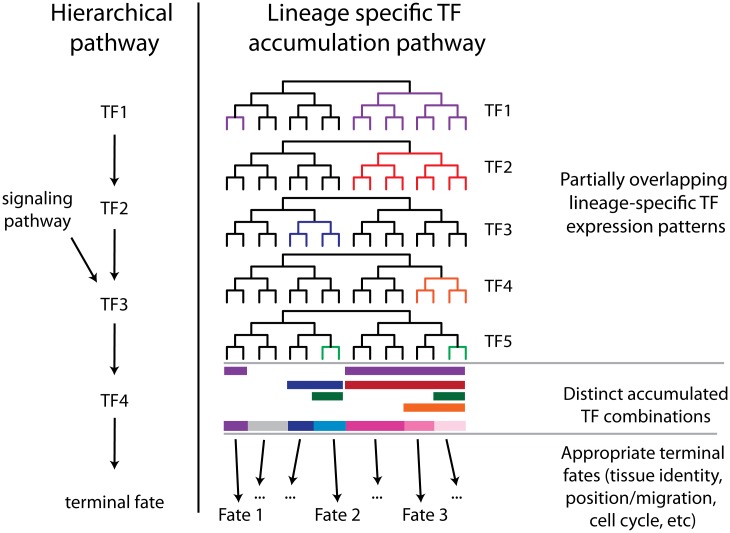
Complementary hierarchical and lineage accumulation models for developmental regulation. A hierarchical model describes the linear order in which early factors regulate later factors to lead to a developmental outcome. In the lineage accumulation model multiple factors are coexpressed across subsets of their expression domain and their expression may be independent of the other factors. The accumulated combination of these factors then results in the appropriate terminal fate. With sufficient redundancy this mechanism could explain the high robustness of *C*. *elegans* development.

The lineage-specific cellular phenotypes and defect penetrance in *ceh-36(-)* and the ABpxp-specific functional interaction between *unc-30* and *ceh-36* are consistent with context-dependent roles for these factors. Each factor has distinct expression outside of the early ABpxp coexpression, suggesting that each may work with other factors in these other lineages; indeed, *unc-30* is a well-established regulator of motor neuron differentiation later in development [49], and *ceh-36* mutants have partially penetrant defects in lineages where *unc-30* is not expressed. Even within ABpxp, most defects were still partially penetrant even in *unc-30; ceh-36* double mutants, consistent with the existence of additional redundant factors. One role of these factors is to directly or indirectly regulate the expression of *mls-2*. Intriguingly, *mls-2* may itself act as a progenitor identity factor, as it regulates the development of lineally-related embryonic cells including glial, excretory and neuronal cells [42,55,57] and is expressed in these cells’ progenitors. In fact, *mls-2* is required for expression of *ceh-36* in the AWC neurons [42], suggesting that *ceh-36* (with *unc-30*) indirectly regulates its own expression later in development. Similarly, *ceh-36* and *unc-30* can bind to the *unc-30* promoter [4], which is intriguing given that the later expression of *unc-30* in GABA-ergic motor neurons occurs in ABpxp-derived cells. We suggest a model in which *C*. *elegans* develops robustly with an invariant lineage because each of many lineage-specific TFs [1], provides a small amount of information to each cell about its lineage history. Combining this information from many TFs allows cells to robustly adopt a fate appropriate to their lineage history (Fig. 9). This model suggests that the expression of each individual factor could be regulated by lineage mechanisms (e.g. [1]) in parallel rather than hierarchically. Another intriguing possibility is raised by our observation of cell cycle and migration defects in cells that nonetheless express appropriate terminal fate markers. This suggests that distinct regulators may modularly control different aspects of each cell’s developmental phenotype (i.e. one factor regulates fate, another, cell cycle, and yet another, migration).

### *ceh-36* and *unc-30* are regulators of ABpxp progenitor identity

Our data suggest that *ceh-36* and *unc-30* act in embryonic progenitor cells to regulate development, which is distinct from their previously characterized role in neuronal terminal differentiation. They are expressed early and transiently in progenitor cells from multiple lineages and these progenitors give rise to varied cell types, similar to multipotent progenitors in other organisms. The migration and cell division defects that we observe occur across these distinct cell types, and while most defects were observed in terminal cells, they were clustered in the lineage suggesting an underlying defect in the common ancestor. Together this strongly suggests that defects occurred in progenitor cells, although it does not rule out additional roles in the subset of terminal cells where *ceh-36* expression persists. Early progenitor factors such as *ceh-36* and *unc-30* may regulate factors important in later progenitor cells, but they could also directly regulate genes expressed in terminal cells by creating stable chromatin alterations, as was recently demonstrated for another factor [58]. Cell division and migration patterns in *unc-30; ceh-36* double mutant embryos do not, however, suggest a switch in fate from ABpxp to its sister ABpxa or any other recognizable sublineage. Thus, other ABpxp factors remain to be discovered or other factors are required to specify alternative progenitor fates.

### Mechanisms ensuring developmental robustness

Gene regulatory networks are generally robust against biological noise and often employ transcription factors (TFs) with overlapping or redundant functions to decrease transcriptional and phenotypic variability [59]. For example, in *C*. *elegans*, redundant pairs of GATA factors regulate intestine development [60], and similar redundancy exists for T-box factors [6,61] and HLH factors [62]. Despite the superficial redundancy of these factors, in some cases the single mutants exhibit decreased robustness in fate determination and partial penetrance phenotypes [60,63]. Our finding of similar redundancy between the more divergent homeodomain factors from the *PITX* and *OTX* classes indicates that redundancy can occur between factors with ancient divergence. Worms, insects, and vertebrates all have *PITX* and *OTX* homologs, indicating these factors diverged prior to the common ancestor of these phyla. This is the first demonstration of a genetic interaction between these factors that could reflect functional redundancy. Since *PITX* and *OTX* factors can bind the same sequence motif this redundancy could reflect regulation of shared targets; consistent with this, a large-scale study of TF binding by yeast 1-hybrid analysis identified binding of *ceh-36* and *unc-30* to highly overlapping sets of promoters [4]. However it is also possible that they work through independent parallel mechanisms. Intriguingly, vertebrate *PITX* and *OTX* homologs have some expression overlap in the pituitary and nervous system, and it will be interesting to determine whether they act together in vertebrates. Our approach of studying robustness across an entire organism at a single-cell level provides the opportunity to sensitively identify cells where each factor or combination of factors plays a role. For example, the overlapping functions of *ceh-36* and *unc-30* in the ABpxp sublineage allowed us to identify their role in regulating *mls-2* expression in the developing excretory system.

### Regulation of lateralization

Previous studies identified *ceh-36* as a regulator of lateral asymmetry for the MI [40] and ASE [38] neurons. The pharyngeal MI neuron is derived from a right lineage, and the left equivalent lineage produces seemingly equivalent cells except for an epithelial cell, e3D, in place of the MI neuron. Mutations in *ceh-36* transform MI into an e3D-like cell, and this asymmetry is driven by asymmetric *ceh-36* expression in the MI progenitors and not those of e3D [40]. Surprisingly, the same phenotype occurs in a truncating mutant in histone H3, likely acting downstream of *ceh-36* [64]. The fact that loss of either an asymmetrically expressed factor (*ceh-36*) or a symmetrically expressed factor (histone H3) leads to the same phenotype underscores that asymmetry in regulatory networks can influence which cells have phenotypes. While we do observe asymmetric CEH-36::GFP expression in MI, we found that most expression in other lineages is L-R symmetric and most penetrant defects were seen in both symmetric pairs. However we did identify defects in lateral identity, such as the migrations of the left intestinal muscle and anal depressor, in cells where *ceh-36* expression is normally L-R symmetric. This suggests that *ceh-36* contributes to the regulation of lateral identity even in cells where it is symmetrically expressed.

### Prospects for comprehensive phenotyping of progenitor TF mutants

Although our approach improved the sensitivity for detection of cellular phenotypes compared to previous studies and methods, it is likely that additional cellular defects remain unidentified. For example we identified many defects in only one or two embryos; further improvements to automated cell tracking methods to increase accuracy and reduce curation time would allow analysis of higher numbers of embryos and more sensitive and reliable identification of lower penetrance defects, In the absence of markers for terminal differentiation, a cell with normal migration and division patterns but altered terminal fate cannot be detected. Repeating lineage tracing with a panel of strains expressing distinct fate markers can increase the power to detect lineage transformations [25], but this approach is labor-intensive. On the other hand, some of the cell position defects we identified were apparent only by lineage tracing and not when scored using a terminal fluorescent marker in larvae, which reflects the high sensitivity of the quantitative methods and possibly the correction of some position defects later in development. The power of future applications of lineage-based phenotyping methods would be increased by new methods to directly assay fate transformation while maintaining throughput; such as by analyzing multiple fate markers simultaneously in different colors.

## Materials and Methods

### Alleles

*ceh-36(ks86)* X

*ceh-36(ky640)* X

*ceh-36(ky646)* X

*ceh-36(ok795)* X

*ceh-37(ok272)* X

*ceh-37(ok642)* X

*unc-30(ok613)* IV

*unc-119(tm4063)* III

*mls-2(cs71)* X

### Reporters

bwIs2[*flp-1*::GFP + pRF4(*rol-6(su1006)*)][65]

nsIs396[*sams-5* 3′::4xNLS-GFP + *lin-15(+)*] V [40]

ntIs1[*lin-15(+); gcy-5*::GFP][38]

sEx14784[*ceh-37*::GFP][46]

ujEx173[CEH-36::GFP *+ unc-119(+)*]

ujEx130[CEH-36::GFP *+ myo-2*::*mCherry + myo-3*::*mCherry*]

oyIs48[*ceh-36* 2KB promoter::GFP][39]

stIs10501[*ceh-36* 5KB promoter::HIS-24-mCherry]*[1]*

ujIs113[*pie-*1::mCherry::H2B *+ unc-119(+);* P*nhr-*2::mCherry::histone *+ unc-119(+)*] II

wgIs108[FKH-4::GFP*+ unc-119(+)*] I [22]

wwIs19[*hlh-6*::GFP *+ unc-119(+)*] X [66]

csIs55[MLS-2::GFP] X [55]

wgIs395[UNC-30::GFP+unc-119(+)]

### Growth conditions and genetics

All strains were grown as previously described [67]. N2 was used as the wild-type reference strain. All manipulations were performed at room temperature (21°C).

Knockout consortium alleles *ceh-36(ok795)* and *unc-30(ok613)* were outcrossed three times. VC579 *ceh-36(ok795)/szT1* hermaphrodites were mated with males carrying an extrachromosomal copy of *ceh-36(+)*::*GFP (ujEx173)*, and F2 progeny were tested for *ok795*, which deletes 406 base pairs of *ceh-36*, by PCR. Additional outcrossing of *ceh-36(ok795)* was with N2 males. *unc-30(ok613)* was outcrossed by mating *unc-30(ok613)* hermaphrodites with N2 males and picking F2 Unc progeny. Combinations of reporters with *ceh-36(-)* were created using a mating strategy that did not produce heterozygous *ceh-36(-)* hermaphrodites at any step or else were verified using PCR.

Combinations of *unc-30(ok613)* and *ceh-36(ok795)* were created using nT1[qIs51](IV;V) to balance *unc-30(ok613)* while testing for *ceh-36(ok795)* by PCR. *unc-30(ok613)*/nT1[qIs51](IV;V); *ceh-36(ok795)* males were mated with *unc-119(tm4063); ceh-36(ok795);* ujEx173[*ceh-36*::GFP *+ unc-119(+)*] hermaphrodites, and F2 Unc progeny with the genotype *unc-30(ok613); ceh-36(ok795);* ujEx173 were isolated. ujEx173 was generated by microparticle bombardment of the CEH-36::GFP Transgeneome fosmid [22] into *unc-119(tm4063)* using methods previously described [22,68]. ujIs113 was generated by co-bombardment of pAA64H2B (pie-1::mCherry-H2B::pie-1UTR)[69] and pJIM20_nhr-2 (nhr-2promoter::HIS-24-mCherry::let-858YTR) into *unc-119(tm4063)*. ujEx130 was generated by injection of the CEH-36::GFP transgeneome fosmid into *ceh-36(ok795*) worms.

### Lethality checks

All strains were grown at 20°C for over two generations before scoring. Young adult hermaphrodites were dissected at room temperature in egg buffer (118mM NaCl, 48mM KCl, 2mM CaCl2, 2mM MgCl2, 25mM HEPES) [70], and embryos with four or more cells were transferred onto NGM plates. Embryos were counted and replaced in the 20°C incubator. Embryonic lethality was determined by counting unhatched embryos on the subsequent two days. Due to a variable rate of larval development for *ceh-36(-)* mutants, L4 hermaphrodites were picked off the NGM plates and counted as survivors for one week following dissection. We observed no L4 lethality or adult sterility. Similar rates of lethality for *ceh-36(ok795)* were obtained by counting eggs laid by free moving *ceh-36(ok795)* hermaphrodites and following their progeny to the L4 stage. To track the presence of the fosmid in rescued animals, we generated *unc-119(tm4063); ceh-36(ok795)* worms that were doubly rescued by the presence of the fosmid and reduced the larval lethality of *ceh-36(ok795*). This allowed us to score absence of the fosmid by the presence of the Unc phenotype.

Lethality checks of *unc-30(ok613); ceh-36(ok795)* double mutants followed a similar protocol. *unc-30(ok613); ceh-36(ok795);* ujEx173[*ceh-36*::GFP *+ unc-119(+)*] young adult hermaphrodites were dissected and embryos counted as described above. Embryonic lethality was scored the next morning. Unhatched embryos were mounted in 20μm beads in egg buffer/methyl cellulose [71] and scored for CEH-36::GFP expression in ASE and AWC neurons. All hatched L1s were examined using a fluorescent dissecting microscope for CEH-36::GFP expression in ASE and AWC neurons (Leica M205FA, Leica Microsystems). CEH-36::GFP expressing and non-expressing L1s were transferred to separate plates, and several larvae were found and transferred the following day. L4 survivors were picked off the NGM plates and counted as survivors for one week following dissection. A similar procedure was used to score survival of *ceh-36(ok795)* worms carrying wwIs19(*hlh-6*::*GFP)*.

### Examination of L1 phenotypes

All strains were grown at 20°C for over two generations before young adult hermaphrodites were dissected at room temperature in egg buffer and embryos with four or more cells were mounted into a solution of 20μm beads in egg buffer/methyl cellulose. Sealed slides containing 10–15 embryos were incubated overnight at 20°C and scored the following morning.

Examination of *unc-30(ok613); ceh-36(ok795)* double mutant phenotypes followed the above protocol except that embryos were also scored for CEH-36::GFP expression in ASE and AWC neurons following DIC examination to exclude rescued animals.

### Live imaging and lineage tracing

We acquired confocal images with a Leica TCS SP5 resonance scanning confocal microscope (67 z planes at 0.5 μm spacing and 1.5 minute time spacing) and generated lineages using StarryNite and AceTree as previously described [20,21,72–75]. Embryos were mounted in egg buffer/methyl cellulose with 20μm beads as spacers [71] and imaged at 22°C using a stage temperature controller (Brook Industries, Lake Villa, IL).

### Detection of deviation in mutant embryos

We updated the 4D reference model of wild-type *C*. *elegans* embryogenesis through the 600-cell stage using eighteen embryos expressing fluorescently tagged histone by tracing four embryos to the comma stage. Deviation of cell-cycle length, division orientation, and anterior-posterior position for eight *ujIs113; ceh-36(ok795)* embryos, one *ujIs113*; *ceh-36(ky646)* and five *ujIs113; unc-30(ok613); ceh-36(ok795); ujEx173(*CEH-36*(+)*::*GFP)* embryos was calculated as previously described [27]. Deviant cell cycle length was defined as beyond three standard deviations and five minutes of the average wild-type cell-cycle length. For position defects, we calculated the expected position of each cell in the embryo based on the overall rotation of the embryo and the wild-type model and scored the distance from the expected position. Cells were considered mispositioned if their mean or maximum distance was more than 3.5 standard deviations greater than the wild-type mean. We also developed a heuristic “neighbor distance” score, consisting of the mean distance of the cell to the 10 cells that are closest to that cell in wild-type embryos, and required 3.5 standard deviation defects in this score as well. Deviant cell position was confirmed by comparison of time-lapse 3D-models for both mutant and wildtype embryos. Defects in all cells identified through statistical analysis mentioned in the text were confirmed by manual retracing of curated lineages. For bootstrap analysis of defective sister pairs, the number of total defective cells (X) and defective sister pairs (Y) were separately counted for each embryo as well as for defined subgroups (e.g. the ABpxp lineage or *ceh-36* expressing versus non-expressing). Cells born before the onset of *ceh-36* expression were not considered. The number of defective sister cells expected by chance was determined by 100,000 iterations of counting sister pairs from (X) randomly picked cells from a defined subgroup. A p-value was calculated by dividing the total number of iterations equal to or greater than the observed value (Y) with 100,000.

### Expression analysis

Mixed-stage embryos were picked into a solution of 10mM sodium azide and 1% methyl cellulose in egg buffer with 25μm beads on top of a glass slide. Coverslips were sealed using petroleum jelly, and embryos became immobilized due to azide and hypoxia. All fluorescent reporters were scored by analyzing confocal GFP and DIC z-stacks of pretzel-stage embryos, which provided a more discrete developmental stage than possible in larvae due to the larval arrest of *ceh-36(-)* mutants. Positional defects and wild-type variation of fluorescent reporters were measured using LASAF software. Single-molecule RNA FISH was performed as previously described [43,76].

## Supporting Information

S1 Table List of strains. (XLSX)

S2 Table Defects observed in each embryo. (XLSX)

S3 Table Classification of defects by 3D visualization. (XLSX)

S4 Table Reporter expression data per cell. (XLSX)

S5 Table Cell position data. (XLSX)

S1 Fig CEH-36::GFP expression. This shows all expressing sublineages. Some nonexpressing cells were not curated to the last time point and are not shown in this figure.
(PNG)

S2 Fig ceh-36(6kb promoter)::H1mCherry expression. This shows all expressing sublineages. Some nonexpressing cells were not curated to the last time point and are not shown in this figure.
(PNG)

S3 Fig ceh-36(2kb promoter):: GFP expression. (PNG)

S4 Fig FKH-4::GFP expression. (PNG)

S5 Fig ceh-37 promoter::GFP expression. This shows all expressing sublineages. Some nonexpressing cells were not curated to the last time point and are not shown in this figure.
(PNG)

S6 Fig UNC-30::GFP expression. This shows all expressing sublineages. Some nonexpressing cells were not curated to the last time point and are not shown in this figure.
(PNG)

S7 Fig Lineage phenotypes in *unc-30(ok650)* single mutant embryos. Defects are displayed as in Figs 4,7.
(PDF)
