# Supplementary figures and images for: The Bicoid Class Homeodomain Factors ceh-36/OTX and unc-30/PITX Cooperate in C. elegans Embryonic Progenitor Cells to Regulate Robust Development
Source: PLoS Genet. 2015 Mar 4;11(3):e1005003. doi: 10.1371/journal.pgen.1005003 (PMC4349592; doi:10.1371/journal.pgen.1005003)

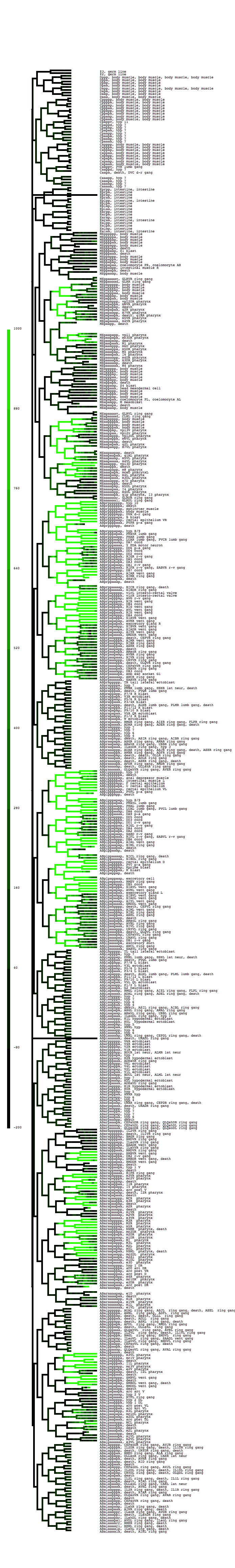

Supplement: S1 Fig — This shows all expressing sublineages. Some nonexpressing cells were not curated to the last time point and are not shown in this figure. (PNG) [file pgen.1005003.s006.png]

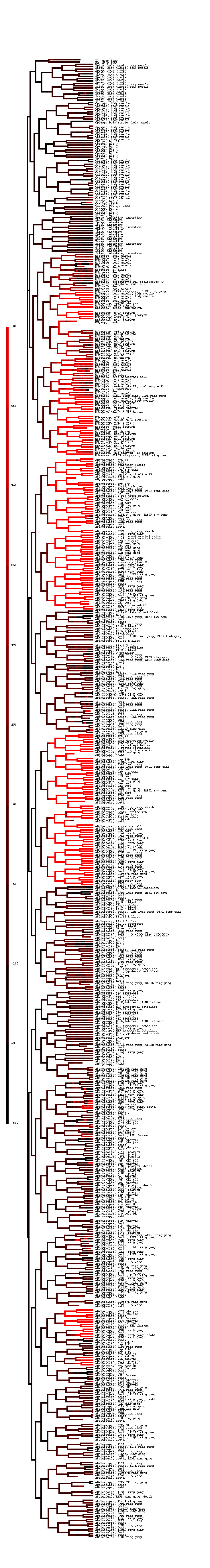

Supplement: S2 Fig — This shows all expressing sublineages. Some nonexpressing cells were not curated to the last time point and are not shown in this figure. (PNG) [file pgen.1005003.s007.png]

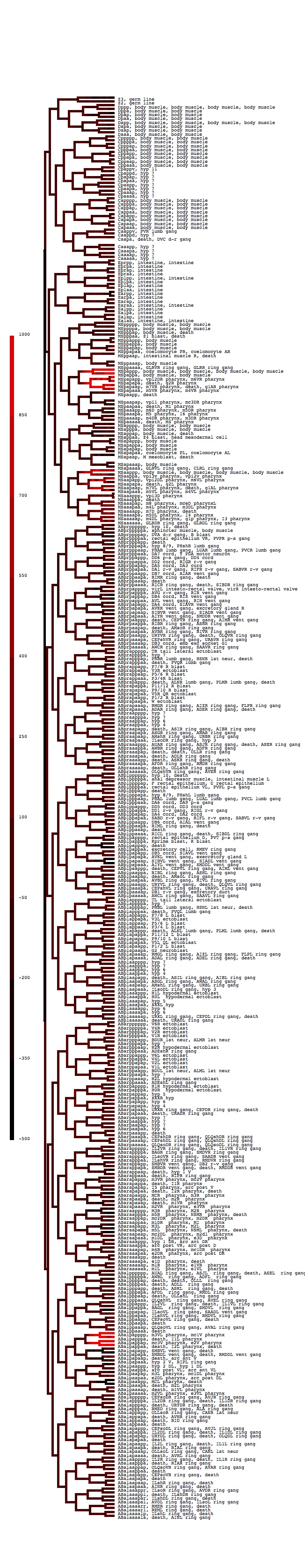

Supplement: S3 Fig — (PNG) [file pgen.1005003.s008.png]

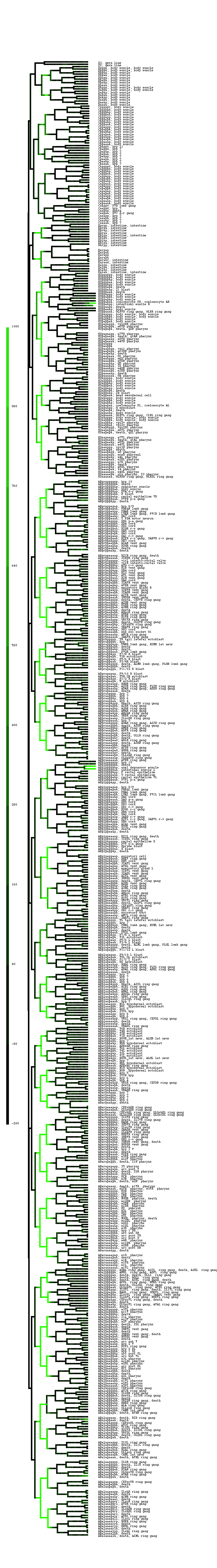

Supplement: S4 Fig — (PNG) [file pgen.1005003.s009.png]

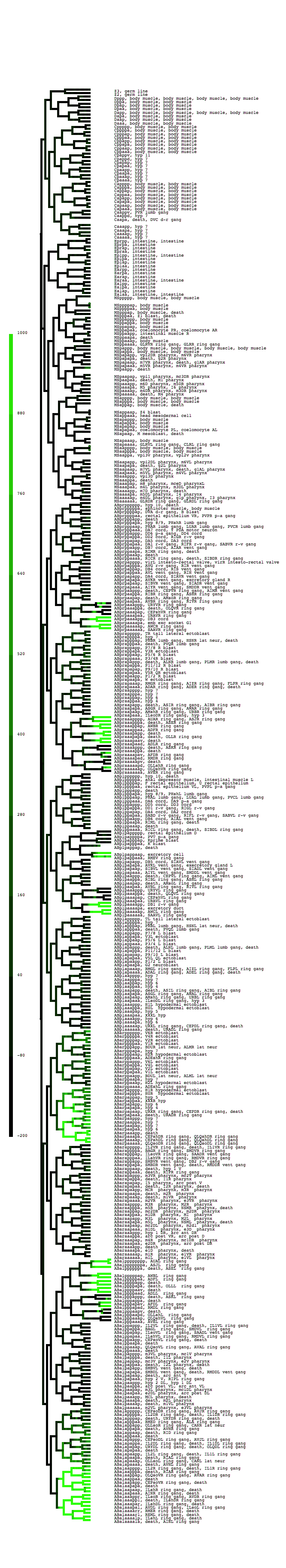

Supplement: S5 Fig — This shows all expressing sublineages. Some nonexpressing cells were not curated to the last time point and are not shown in this figure. (PNG) [file pgen.1005003.s010.png]

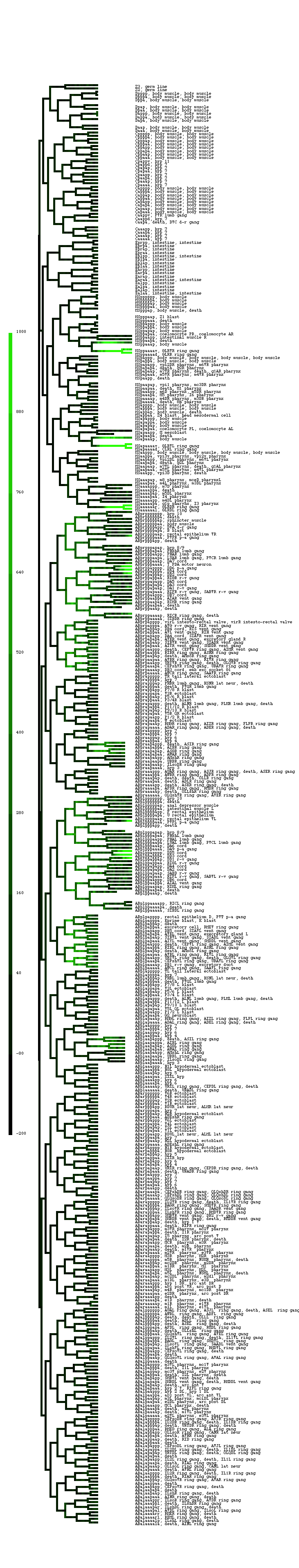

Supplement: S6 Fig — This shows all expressing sublineages. Some nonexpressing cells were not curated to the last time point and are not shown in this figure. (PNG) [file pgen.1005003.s011.png]

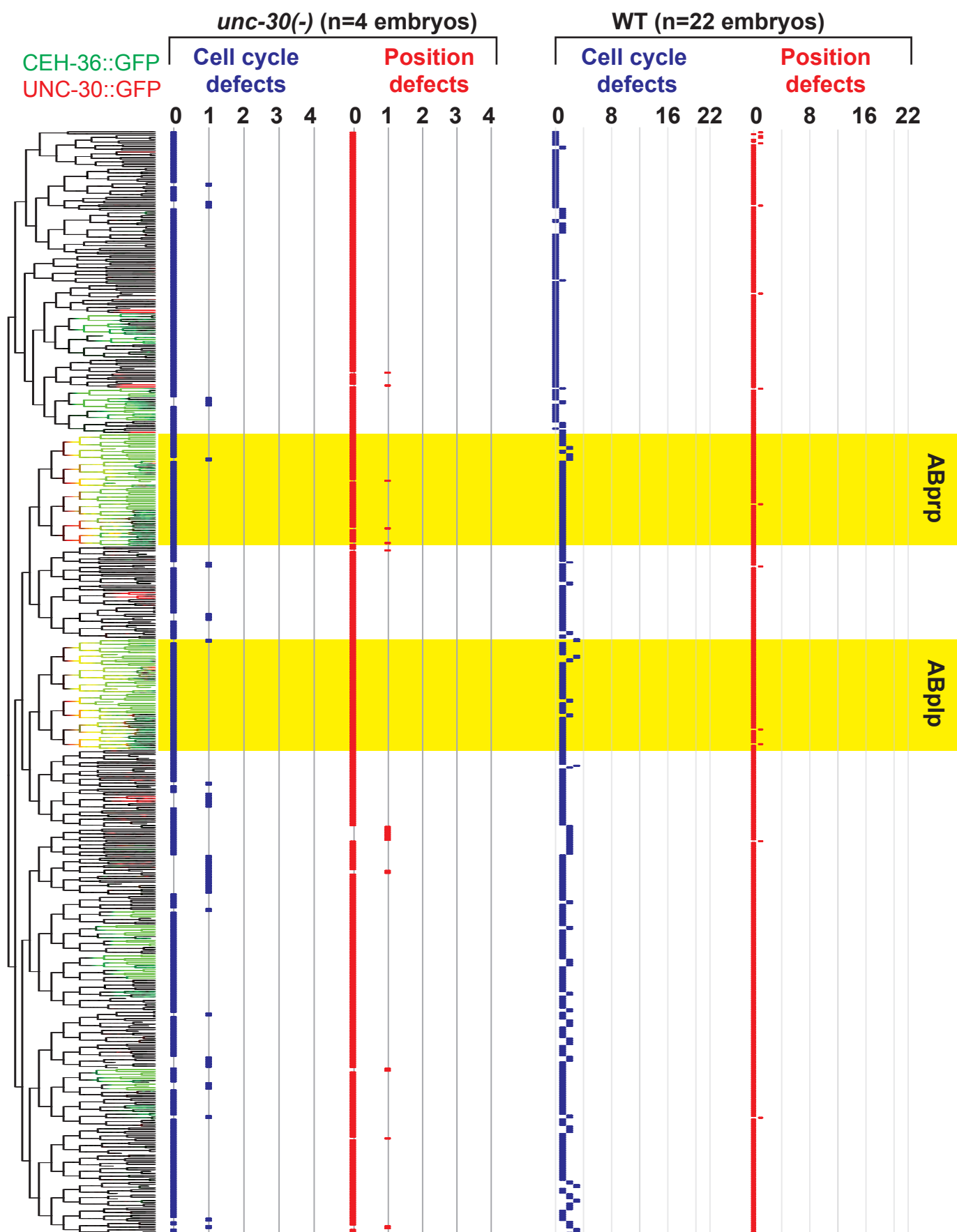

Supplement: S7 Fig — Defects are displayed as in Figs 4,7. (PDF) [file pgen.1005003.s012.pdf]
